# Alkaloid Composition and Biological Activities of the Amaryllidaceae Species *Ismene amancaes* (Ker Gawl.) Herb.

**DOI:** 10.3390/plants11151906

**Published:** 2022-07-22

**Authors:** Marilú Roxana Soto-Vásquez, Cecilia Anataly Rodríguez-Muñoz, Luciana R. Tallini, Jaume Bastida

**Affiliations:** 1 Facultad de Farmacia y Bioquímica, Universidad Nacional de Trujillo, Av. Juan Pablo II, Trujillo 13011, Peru; anace_acua@hotmail.com; 2 Programa de Pós-Graduação em Ciências Farmacêuticas, Faculdade de Farmácia, Universidade Federal do Rio Grande do Sul, Av. Ipiranga 2752, Porto Alegre 90610-000, RS, Brazil; lucianatallini@gmail.com; 3 Departament de Biologia, Sanitat i Medi Ambient, Facultat de Farmàcia i Ciències de l’Alimentació, Universitat de Barcelona, Av. Joan XXIII #27–31, 08028 Barcelona, Spain; jaumebastida@ub.edu

**Keywords:** Amaryllidaceae, alkaloids, Alzheimer’s disease, *Ismene amancaes*, malaria, *Plasmodium falciparum*

## Abstract

Natural products have always played a significant role in the search for new drugs. One of the most relevant alkaloid-containing plant groups is the Amaryllidaceae family, a source of exclusive structures with a wide variety of pharmacological activities. The aim of this work was to determine the alkaloid composition and biological potential of an extract from the bulbs of an endemic Peruvian Amaryllidaceae species *Ismene amancaes* (Ker Gawl.) Herb. The alkaloid profiling was carried out by GC-MS, which revealed the presence of 13 compounds, 2 of them unidentified. The plant extract was found to contain high amounts of lycoramine, a galanthamine-type alkaloid. The extract also presented low inhibitory potential against the enzymes AChE and BuChE, with IC_50_ values of 14.6 ± 0.6 and 37.6 ± 1.4 μg·mL^−1^, respectively, and good to moderate inhibitory activity against the protozoan *Plasmodium falciparum* strain FCR-3 (chloroquine-resistant), with IC_50_ values of 3.78 ± 0.3 μg·mL^−1^. This is the first report of the alkaloid profile of a plant of the *Ismene* genus, which could be an interesting source of bioactive compounds.

## 1. Introduction

Malaria is caused by infection with intracellular parasites of the genus *Plasmodium*, which are transmitted to people through the bite of female *Anopheles* mosquitoes, with *P. falciparum* being the most lethal [1]. The World Health Organization estimated an increase in malaria cases from 227 million in 2019 to 241 million in 2020 [2]. Although significant success has been achieved in malaria control through preventive interventions such as insecticide-treated nets, vector management, and more effective treatment with artemisinin-combined drug therapy, these gains are threatened by the emergence of drug resistance [3]. The first antimalarial drug used was quinine, which was isolated from *Cinchona* species (Rubiaceae) by Pelletier and Caventou in 1820 [4,5]. Preparations of the bark of this tree, known as “quina”, were used by the Peruvian indigenous population to treat fevers and were introduced into Europe at the beginning of the XVIII century [4]. At present, antimalarial drugs such as derivatives of quinoline and artemisinin, as well as antifolates, are being used [5]. However, it is essential to search for new options to improve the treatment of the disease [6].

On the other hand, dementia is a syndrome characterized by deterioration in cognitive function, and is currently the seventh leading cause of death among all diseases [7]. Alzheimer’s disease is the most common form of this disorder, being responsible for 60–70% of dementia cases [7]. Galanthamine, isolated for the first time from the species *Galanthus woronowii* (Amaryllidaceae) in the 1950s, is one of the drugs used for the palliative treatment of mild to moderate Alzheimer’s disease [8,9]. This alkaloid, approved by the FDA in 2001, has an inhibitory effect on the enzyme acetylcholinesterase (AChE), increasing the levels of acetylcholine (ACh) in the inter-synaptic space, which is important for cognitive functions [10,11]. This fact has attracted the attention of researchers to the Amaryllidaceae family, which could be an interesting source for new bioactive molecules [12]. Together with galanthamine, donepezil and rivastigmine are the only cholinesterase inhibitor drugs approved by the FDA for the clinical treatment of Alzheimer’s disease [13]. The neurotransmitter ACh can also be hydrolyzed by butyrylcholinesterase (BuChE) [14]. In patients with Alzheimer’s disease, AChE activity in the brain decreases, while that of BuChE increases [10]. Therefore, an interesting therapeutic response could be generated by cholinesterase inhibitors that suppress both enzymes [15].

Natural products represent a valuable source of bioactive agents for evaluation in the search for new medicines [16]. The bioactivity of alkaloids, metabolites found mainly in plants, can be harnessed to develop new therapeutic strategies [17]. The Amaryllidaceae plant family, specifically the subfamily Amaryllidoideae, contains an exclusive group of alkaloids known as Amaryllidaceae alkaloids, which have revealed remarkable biological activities, including antiparasitic, anti-cholinesterase, antifungal, antiviral, antibacterial, antiproliferative, cytotoxic, and psychopharmacological effects [18,19].

New information about the chemical profiles and biological potential of Amaryllidoideae plants, especially previously unstudied species, has been published in the last decade [20]. This subfamily comprises more than 800 species classified into 59 genera, which are found in different climate conditions, mostly in South Africa, South America, and the Mediterranean region [18]. The Amaryllidoideae species *Ismene amancaes* (Ker Gawl.) Herb. is endemic to Peru, found in the central coast and northern highlands [21], and is known as an ornamental plant owing to the beauty of its yellow flowers [22].

Therefore, the aim of this work was to evaluate the chemical and biological potential of *I. amancaes* collected in northern Peru. The alkaloid profile of a bulb extract of this species was obtained by gas chromatography-mass spectrometry (GC-MS). Antiplasmodial and cholinesterase—acetylcholinesterase (AChE) and butyrylcholinesterase (BuChE)—inhibitory activities of the plant extract were investigated.

## 2. Results and Discussion

### 2.1. Alkaloid Profiling

**Figure 1:**
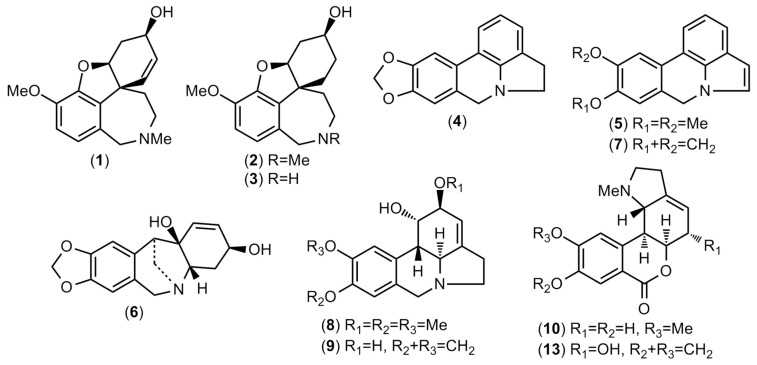
Amaryllidaceae alkaloids identified in *Ismene amancaes* bulbs by GC-MS.

The bulb extract of *Ismene amancaes* was evaluated by GC-MS and 11 Amaryllidaceae alkaloids were identified (Figure S1). These alkaloids are described in Table 1; the amounts are presented as mg of galanthamine (GAL), which was related to g of alkaloid extract (mg GAL·g^−1^ AE). All of the structures identified in this plant are depicted in Figure 1.

The Amaryllidaceae alkaloids have usually been classified in nine skeleton types, four of which were observed in this extract. Galanthamine, lycoramine, and norlycoramine, described in Table 1, all contain the galanthamine-type scaffold. Galanthamine is currently being obtained on an industrial scale from Amaryllidaceae species such as *Narcissus* cv *Carlton*, *Lycoris radiata*, and *Leucojum aestivum*, owing to the low yield and high cost of its synthesis [30]. The species *N*. cv *Carlton* and *L*. *radiata* are the basis of the industries in Europe and China, respectively, although both species also present significant quantities of other alkaloids [30,31]. Moreover, the biomass for galanthamine extraction from *L. aestivum* is gathered from nature because there is no any efficient agricultural technology for its large-scale cultivation [8]. Then, there is an urgent need to find new sources of this valuable pharmaceutical product [30].

As shown in Table 1, lycoramine (2) was the major alkaloid detected in the *I. amancaes* bulb extract (132.2 mg GAL·g^−1^ AE). The structure of this alkaloid is very similar to that of galanthamine (**1**), as shown in Figure 1, suggesting that *I. amancaes* could be of interest to pharmaceutical companies as a source of lycoramine for a semi-synthetic approach to galanthamine production.

Five alkaloids containing the lycorine-type skeleton were identified in the *I. amancaes* bulb extract: anhydrolycorine (**4**), assoanine (**5**), 11,12-dehydroanhydrolycorine (**7**), galanthine (**8**), and lycorine (**9**). Among them, lycorine (**9**) was detected in high amounts (51.2 mg GAL·g^−1^ AE), followed by galanthine (**8**) (22.5 mg GAL·g^−1^ AE). 8-*O*-demethylhomolycorine (**10**) and hippeastrine (**13**), both containing a homolycorine-type scaffold, and pancratinine C (**6**), a montanine-type alkaloid, were also identified (Table 1). Two compounds were detected in this plant extract that could not be identified, although their mass spectra were evaluated using the NIST 05 Database and compared to data in the literature.

### 2.2. AChE and BuChE Inhibitory Activity

**Figure 2:**
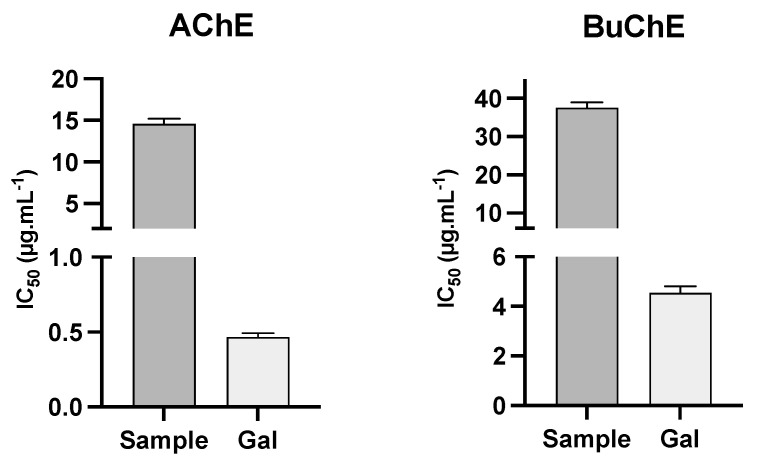
AChE and BuChE inhibitory activity of *Ismene amancaes*. Sample: alkaloid extract of *I. amancaes* bulbs; Gal: galanthamine (reference compound).

The alkaloid extract of *Ismene amancaes* bulbs presented low inhibitory activity against AChE and BuChE, with IC_50_ values of 14.6 ± 0.6 and 37.6 ± 1.4 μg·mL^−1^, respectively, while galanthamine (the reference control) showed IC_50_ values of 0.47 ± 0.03 and 4.54 ± 0.27 μg·mL^−1^, respectively (Figure 2). The anti-cholinesterase activity of the plant extract could be due to its high concentration of lycoramine, which has a galanthamine-type structure.

According to the literature, the results of molecular docking experiments carried out with lycoramine suggest that it has potential application as an AChE inhibitor [32]. Conversely, a recent study reported low activity for lycoramine in vitro against AChE (IC_50_ values of 42.48 ± 1.66 μg·mL^−1^) and no BuChE inhibitory potential [33].

The presence of high amounts of the alkaloids lycorine (**9**), hippeastrine (**13**), and galanthine (**8**) could also be responsible for the cholinesterase inhibitory activity of the extract (Figure 2). However, some authors have described that lycorine is inactive against cholinesterase in vitro, with IC_50_ values higher than 50 μg·mL^−1^ [33,34]. The same authors report that galanthine has in vitro AChE inhibitory activity, with IC_50_ values of 1.96 ± 0.01 μg·mL^−1^, and a different mode of interaction with AChE than that observed for galanthamine in molecular dynamics experiments [33]. A search of the literature revealed only one scientific publication about the cholinesterase potential of other *Ismene* species, which reported that an ethanol extract of *Ismene festalis* was active against AChE and BuChE in a TLC bioautographic assay [35].

### 2.3. Antiplasmodial Activity

**Figure 3:**
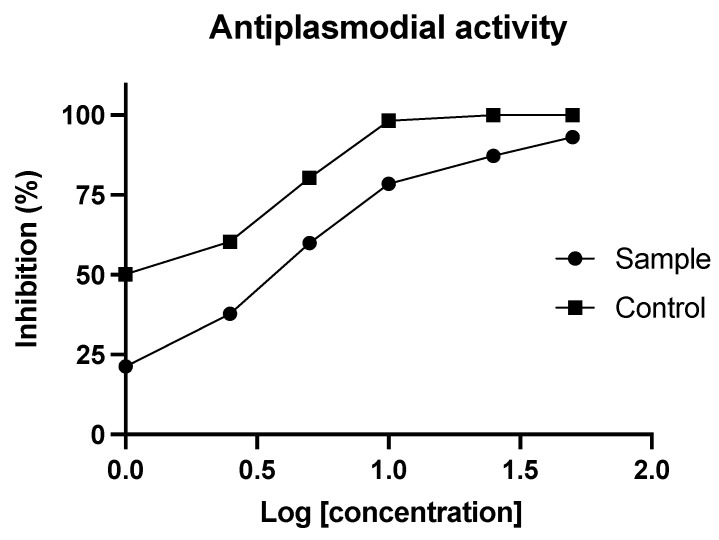
In vitro antiplasmodial activity of the alkaloid extract from bulbs of *Ismene amancaes*.

As shown in Figure 3, the alkaloid extract from the bulbs of *Ismene amancaes* at concentrations of 1, 2.5, 5, 10, 25, and 50 μg·mL^−1^ showed in vitro inhibitory activity of 20.5 ± 0.3% to 70.1 ± 0.5% against the protozoan *P. falciparum* (strain FCR-3, chloroquine-resistant). The highest percentage of parasite inhibition by the plant extract was obtained at the concentration of 50 μg·mL^−1^, with an IC_50_ value of 3.78 ± 0.3 μg·mL^−1^, while the reference drug, chloroquine, presented IC_50_ values of 0.06 ± 0.2 μg·mL^−1^. According to the criteria of the Research Initiative on Traditional Antimalarial Methods, validated by Willcox and co-authors [36], the IC_50_ values obtained for *I. amancaes* extract may be classified as good to moderate.

According to the literature, among the different Amaryllidaceae alkaloids, the highest antiplasmodial activity is displayed by those with haemanthamine/crinine- and lycorine-type skeletons [37,38]. A study found that two different extracts of *Crinum bulbispermum* (Amaryllidaceae), a plant that usually contains haemanthamine/crinine-type alkaloids and has probably been used in traditional medicine to treat malaria, showed activity against the same strain of *P. falciparum* as used herein (FCR-3), with IC_50_ values of 0.38 and 0.08 μg·mL^−1^ [37,39].

As shown in Table 1, lycoramine (**2**), lycorine (**9**), hippeastrine (**13**), and galanthine (**8**) were the predominant alkaloids detected in the *I. amancaes* bulb extract. According to the literature, the alkaloid galanthine, isolated from *Zephyranthes citrina,* presented interesting antiplasmodial activity against the strain K1 (stage IEF), with IC_50_ values of 0.2 μg·mL^−1^ [40]. Among the numerous publications about the antiplasmodial activity of lycorine, one study reported IC_50_ values of 0.029 μg·mL^−1^ against the FCR-3 strain of *P. falciparum* [39], but others found no activity for lycorine against the strains NF54 and K1 [29].

A study described that lycoramine (**2**) and hippeastrine (**13**), isolated from *Narcissus poeticus* (Amaryllidaceae), did not display any discernible activity against the hepatic stage of *Plasmodium berghei* [41]. Despite that, interesting results were recently obtained for the alkaloid hippeastrine (**13**) against Chagas disease, which is caused by the protozoan *Trypanosoma cruzi* [42].

## 3. Materials and Methods

### 3.1. Plant Material

The bulbs of *Ismene amancaes* (Ker Gawl.) Herb. were collected during the flowering period in the surroundings of the town Pagash Alto, Salpo District (2160 masl), Otuzco Province, La Libertad Region (Peru) in 2021. The species was authenticated by Prof. Alan Meerow from the Agricultural Research Service, United State Department of Agriculture (USA), and a specimen voucher with registration number 63331 HUT was deposited at the Herbarium Truxillense of the National University of Trujillo (HUT), Peru.

### 3.2. Alkaloid Extraction

The bulbs were washed, disinfected and cut into thin slices, and then dried in a forced convection oven at 40 °C for 72 h. Once the plant material was completely dry, it was ground in a rotary blade mill. About 1 g of dry powder material was macerated with methanol for 72 h at room temperature, changing the solvent daily (3 × 100 mL), and applying 20 min of ultrasonic baths, eight times per day. Subsequently, the methanolic extract was filtered and evaporated to dryness under reduced pressure using a rotary evaporator at a temperature of 40 °C. The crude extract obtained (340 mg) was acidified to pH 3 with H_2_SO_4_ (2%, *v*/*v*). The neutral material was removed with Et_2_O. The acidic aqueous phase was subjected to basification with NH_4_OH (25%, *v*/*v*) until a pH of 10 was reached. The alkaloids were then extracted through the repeated use of EtOAc, and the organic solvent was evaporated under reduced pressure in the rotary evaporator at a temperature of 40 °C to obtain the alkaloid extract (AE) (16 mg).

### 3.3. GC-MS Analysis

The AE was analyzed using a GC-MS apparatus (Agilent Technologies 6890 N coupled with MSD5975 inert XL; Santa Clara, CA, USA) operating in the electron ionization (EI) mode at 70 eV. A Sapiens-X5 MS column (30 m × 0.25 mm i.d., film thickness 0.25 µm) was used. The temperature gradient was as follows: 12 min at 100 °C, 100–180 °C at 15 °C·min^−1^, 180–300 °C at 5 °C·min^−1^, and 10 min hold at 300 °C. The injector and detector temperatures were 250 and 280 °C, respectively, and the flow-rate of carrier gas (He) was 1 mL·min^−1^. One milligram of each total alkaloid was dissolved in 0.5 mL of MeOH:CHCl_3_ (1:1, *v*/*v*) and 1 µL was injected using the split-less mode. Codeine (50 µg·mL^−1^) was used as an internal standard.

### 3.4. Alkaloid Identification and Quantification

The chromatogram was analyzed using the software AMDIS 2.64 (NIST, MD, USA). The alkaloids were identified by comparing their GC-MS spectra and Kovats retention indices (RIs) with the library database developed by the Natural Products Group of the University of Barcelona (Spain). The database includes Amaryllidaceae alkaloids previously isolated and identified using different spectroscopic and spectrometric techniques, such as nuclear magnetic resonance (NMR) and mass spectrometry (MS). The alkaloids have also been evaluated using the NIST 05 Database (NIST, MD, USA) [43] and compared to data from the literature [23,24,25,26,27,28,29]. The alkaloid structures were obtained using the software ChemDraw 12.0 (PerkinElmer Informatics, MA, USA).

A calibration curve of galanthamine (10, 20, 40, 60, 80, and 100 µg·mL^−1^) was applied to quantify each single constituent detected in the chromatogram, using codeine (50 µg·mL^−1^) as the internal standard. Peak areas were manually obtained, considering the selected ions for each compound (usually the base peak of their MS, i.e., *m/z* at 286 for galanthamine and 299 for codeine). The ratio between the values obtained for galanthamine and codeine in each solution was plotted against the corresponding concentration of galanthamine to obtain the calibration curve and its equation (y = 0.023x − 0.2535; R^2^ = 0.9991). All data were standardized to the area of the internal standard (codeine), and the equation obtained for the calibration curve of galanthamine was used to calculate the amount of each alkaloid. The single alkaloid content in the extract was quantified and reported as mg GAL·g^−1^ AE. As the peak area not only depends on the corresponding alkaloid concentration, but also on the intensity of the mass spectra fragmentation, the quantification is not absolute.

### 3.5. AChE and BuChE Inhibitory Activity

Cholinesterase inhibitory activities were determined according to Ellman and co-workers [44] with some modifications by López and co-workers [45]. Stock solutions with 518U of AChE from *Electrophorus electricus* (Merck, Darmstadt, Germany) and BuChE from equine serum (Merck, Darmstadt, Germany), respectively, were prepared and kept at −20 °C. Acetylthiocholine iodide (ATCI), *S*-butyrylthiocholine iodide (BTCI), and 5,5′-dithiobis(2-nitrobenzoic acid) (DTNB) were obtained from Merck (Darmstadt, Germany). Fifty microliters of AChE or BuChE (both enzymes used at 6.24 U) in phosphate buffer (8 mM K_2_HPO_4_, 2.3 mM NaH_2_PO_4_, 0.15 NaCl, pH 7.5) and 50 μL of the sample dissolved in the same buffer were added to the wells. The plates were incubated for 30 min at room temperature. Then, 100 μL of the substrate solution (0.1 M Na_2_HPO_4_; 0.5 M DTNB; and 0.6 mM ATCI or 0.24 mM BTCI in Millipore water, pH 7.5) was added. These reagents were obtained from Merck (Darmstadt, Germany). After 10 min, the absorbance was read at 405 nm in a Labsystem microplate reader (Helsinki, Finland). Enzymes activities were calculated as a percentage compared with a control using a buffer without any inhibitor. Galanthamine served as a positive control. In the first step, the activity of the sample towards both enzymes was assessed at 10, 100, and 200 μg·mL^−1^. Then, the calibration curves of the AE (1, 10, 15, 25, and 50 μg·mL^−1^) and (1, 10, 25, 50, and 75 μg·mL^−1^) were applied to obtain IC_50_ values against AChE and BuChE, respectively. The cholinesterase inhibitory data were analyzed with Prism 9 software (GraphPad, CA, USA).

### 3.6. Antiplasmodial Inhibitory Activity

The antiplasmodial activity of the AE of *I. amancaes* bulbs was carried out in vitro using the strain FCR3 (chloroquine resistant) of *P. falciparum*. The strain was cultured in RPMI 1640 medium supplemented with 10% human serum and 4% hematocrit, which was obtained by adding 200 µL of total red blood cells in 4.5 mL of RPMI 1640 and 0.5 mL of serum or plasma (blood group 0, Rh+). Incubation was carried out at 37 °C in a 5% O_2_ 6% gas mixture atmosphere of CO_2_ and balanced N_2_, as described by Trager and co-authors [46], with some modifications. The tests for the antiplasmodial activity of AE (1.0, 2.5, 5.0, 10.0, 25.0, and 50.0 µg·mL^−1^ dissolved in DMSO) were carried out in flat bottom 96-well plates for each AE in triplicate. Chloroquine diphosphate (10 to 1000 nM) was used as a control of the test. The cultures were synchronized with a parasitaemia and a hematocrit of 1 and 2%, respectively. These were dispensed in a volume of 100 µL in 96-well plates in duplicate, 100 µL of the total alkaloids were added, and finally they were incubated at 37 °C for 48 h. After this incubation time, the upper phase of the culture was eliminated to obtain a smear of the sediment from each well, which was fixed with methanol and stained with Giemsa. The plates were observed under the microscope with a 100x immersion lens, counting uninfected and infected red blood cells, in order to obtain the percentage of inhibition [47].

### 3.7. Statistical Analysis

The results were processed using the statistical program SPSS v. 23 (SPSS Inc., IL, USA) and expressed as the arithmetic median ± standard deviation. The relationship between the groups was determined using a one-way ANOVA test, in which *p* < 0.05 was considered statistically significant.

## 4. Conclusions

This work is the first to report the alkaloid profile of a plant of the genus *Ismene* Salisb. ex Herb., represented herein by *I. amancaes*. High concentrations of lycoramine, a galanthamine-type alkaloid, were detected in this species, suggesting it could be evaluated by pharmaceutical companies for a semi-synthetic approach to galanthamine production. The biological potential of this species was also determined, and the alkaloid extract of *I. amancaes* bulbs showed low activity against AChE and BuChE enzymes, and good to moderate antiplasmodial activity against the protozoan *P. falciparum* (strain FCR-3). The presence of the alkaloids lycoramine, lycorine, galanthine, and hippeastrine, detected in high amounts in the *I. amancaes* bulb extract, could account for the biological results obtained here; however, more experiments are necessary to verify this. These findings support the potential of Amaryllidaceae species as a valuable source of bioactive alkaloids.

## Figures and Tables

**Table 1:** GC-MS alkaloid profiling of *Ismene amancaes* bulbs collected in Peru.

Alkaloid	[M^+^]	MS	RI ^1^	Amount ^2^	TIC (%) ^3^	References
galanthamine (**1**)	287 (79)	286 (100), 270 (14), 244 (22), 216 (24)	2430.5	6.7	0.9	[23]
lycoramine (**2**)	289 (72)	288 (100), 202 (12), 187 (10)	2463.5	132.2	39.1	[23]
norlycoramine (**3**)	275 (83)	274 (100), 188 (16)	2495.1	10.3	2.9	[24]
anhydrolycorine (**4**)	251 (49)	250 (100), 192 (10)	2543.5	8.6	1.3	[25]
assoanine (**5**)	267 (52)	266 (100), 250 (21)	2611.9	6.3	0.4	[26]
pancratinine C (**6**)	287 (95)	203 (52), 188 (58), 174 (100), 148 (43)	2622.2	6.4	0.6	[27]
11,12-dehydroanhydrolycorine (**7**)	249 (68)	248 (100), 190 (22)	2645.6	6.3	0.4	[25]
galanthine (**8**)	317 (29)	284 (13), 268 (16), 243 (94), 242 (100)	2736.2	22.5	7.9	[28]
lycorine (**9**)	287 (35)	268 (24), 250 (15), 227 (75), 226 (100)	2796.4	51.2	15.8	[24]
8-*O*-demethylhomolycorine (**10**)	301 (-)	109 (100), 108 (22)	2844.2	10.7	1.9	[29]
UI ^4^ (**11**)	297 (60)	296 (100), 280 (17)	2852.1	9.3	1.4	-
UI ^4^ (**12**)	279 (76)	278 (100), 262 (12)	2859.3	6.7	0.8	-
hippeastrine (**13**)	315 (-)	125 (100), 96 (40)	2928.5	33.7	12.6	[24]

^1^ RI: Kovats retention index; ^2^ values expressed as mg GAL·g^−1^ AE; ^3^ TIC: total ion current; ^4^ UI: unidentified compound.

## Data Availability

Not applicable.

## Supplementary Materials

The following are available online at https://www.mdpi.com/article/10.3390/plants11151906/s1, Figure S1: GC chromatogram of the alkaloid extract of Ismene amancaes bulbs collected in Peru.
