# Supplementary material for: Alkaloid Composition and Biological Activities of the Amaryllidaceae Species Ismene amancaes (Ker Gawl.) Herb
Source: Plants (Basel). 2022 Jul 22;11(15):1906. doi: 10.3390/plants11151906 (PMC9331871; doi:10.3390/plants11151906)
Supplement: Supplementary file 1 [file plants-11-01906-s001.zip › plants-1818059-supplementary.pdf]

plants

Article

Alkaloid Composition and Biological Activities of the Amaryllidaceae Species *Ismene amancaes* (Ker Gawl.) Herb.

Marilú Roxana Soto-Vásquez [1,*], Cecilia Anataly Rodríguez-Muñoz [1], Luciana R. Tallini [2,3] and Jaume Bastida [3]

[1] Facultad de Farmacia y Bioquímica, Universidad Nacional de Trujillo, Av. Juan Pablo II, Trujillo 13011, Peru; anace_acua@hotmail.com
[2] Programa de Pós-Graduação em Ciências Farmacêuticas, Faculdade de Farmácia, Universidade Federal do Rio Grande do Sul, Av. Ipiranga 2752, Porto Alegre 90610-000, RS, Brazil; lucianatallini@gmail.com
[3] Departament de Biologia, Sanitat i Medi Ambient, Facultat de Farmàcia i Ciències de l'Alimentació, Universitat de Barcelona, Av. Joan XXIII #27–31, 08028 Barcelona, Spain; jaumebastida@ub.edu
* Correspondence: msoto@unitru.edu.pe

Supplementary Materials

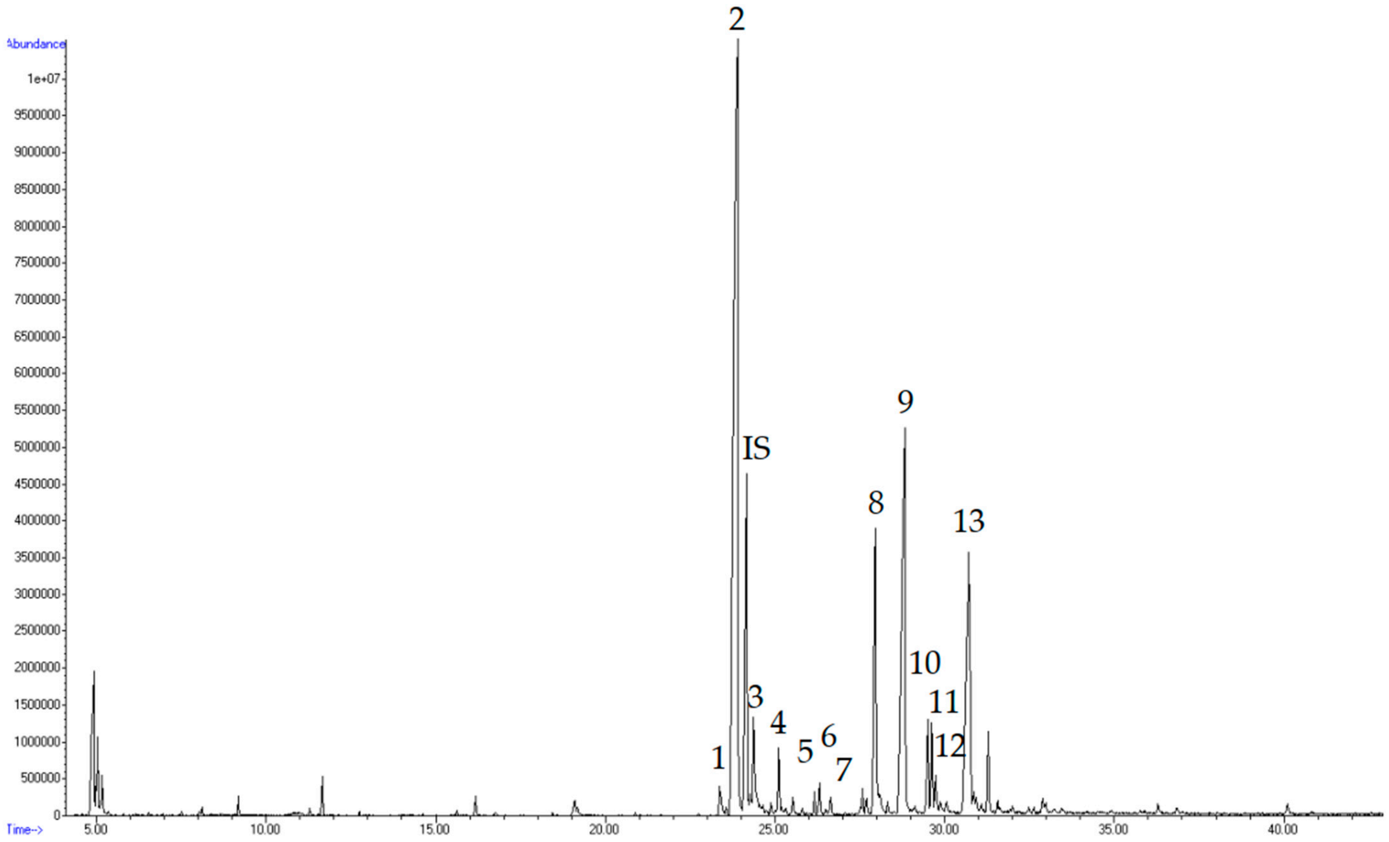

Figure S1. GC chromatogram of the alkaloid extract of Ismene amancaes bulbs collected in Peru. IS: internal standard (codeine).
